# The impact of emotionally challenging situations on medical students’ professional identity formation

**DOI:** 10.1007/s10459-023-10229-8

**Published:** 2023-05-15

**Authors:** Annalena Lönn, Maria Weurlander, Astrid Seeberger, Håkan Hult, Robert Thornberg, Annika Wernerson

**Affiliations:** 1 Division of Renal Medicine, Department of Clinical Science, Intervention and Technology (CLINTEC), Karolinska Institutet, Alfred Nobels Allé 8, 141 52 Huddinge, Stockholm, Sweden; 2 Department of Education, Stockholm University, Stockholm, Sweden; 3 Department of Behavioural Sciences and Learning, Linköping University, Linköping, Sweden

**Keywords:** Emotionally challenging situations, Medical students, Narrative data, Grounded theory, Self-awareness, Professional identity formation

## Abstract

In their interactions with patients and health care professionals during work-based learning, medical students are known to experience emotionally challenging situations that can evoke negative feelings. Students have to manage these emotions. Students learn and develop their professional identity formation through interactions with patients and members of the healthcare teams. Earlier studies have highlighted the issues involved with processing emotionally challenging situations, although studies concerning learning and professional identity formation in response to these experiences are rare. In this study, we explored medical students’ experiences of emotionally challenging situations in work-based learning, and the impact these experiences had on forming medical students’ professional identities. We conducted an analysis of narrative data (*n* = 85), using a constructivist grounded theory approach. The narratives were made up of medical students’ reflective essays at the end of their education (tenth term). The analysis showed that students’ main concern when facing emotionally challenging situations during their work-based education was the struggle to achieve and maintain a professional approach. They reported different strategies for managing their feelings and how these strategies led to diverse consequences. In the process, students also described arriving at insights into their own personal needs and shortcomings. We consider this development of self-awareness and resulting self-knowledge to be an important part of the continuously ongoing socialization process of forming a professional identity. Thus, experiencing emotionally challenging situations can be considered a unique and invaluable opportunity, as well as a catalyst for students’ development. We believe that highlighting the impact of emotions in medical education can constitute an important contribution to knowledge about the process of professional identity formation. This knowledge can enable faculty to provide students with more effective and sufficient support, facilitating their journey in becoming physicians.

## Introduction

### Emotionally challenging situations

The clinical reality to which medical students are exposed during work-based learning is complex, and stakes can be high in an environment that is sometimes intense, unpredictable, and demanding (O'Flynn et al., 2014). An essential part of students’ skills training takes place in clinical settings where students interact with patients, physicians, other healthcare professionals, and peers (Bandini et al., 2017; Bleakley, 2006; Dornan, 2012). Students have been found to experience difficult situations during work-based learning that will have a significant impact on them and produce strong emotions (Helmich et al., 2014; Monrouxe et al., 2015; Weurlander et al., 2018), which in turn play an important part in identity formation (Dornan et al., 2015). Such situations include confronting patients’ illness, suffering, and death; witnessing unprofessional behavior among fellow healthcare professionals; perceived dilemmas regarding patient treatment; relating to patients as diagnoses rather than as human beings (dehumanization), and being uncomfortable in situations where patients are used solely for students’ education. Other studies have described encounters with patients who are considered “difficult”—in the sense of being angry, noncompliant, or overly talkative—as challenging (Shapiro et al., 2016), with students reporting negative feelings such as frustration and anxiety. Thus, students have to relate to a range of difficult and challenging situations during their work-based learning and the following negative emotions, which have been described as overwhelming, confusing, and daunting (Shacklady et al., 2009). Students have also reported an overarching feeling of uncertainty related to emotionally challenging situations, including doubts about their own abilities to endure and uncertainty about whether they want to be part of a medical culture perceived as negative (Weurlander et al., 2019). Experiences, including emotional challenges, during work-based education, have the potential to have a major impact on medical students’ professional becoming.

This study was performed before the beginning of the COVID-19 pandemic.

### Emotions

Emotions are known to play an important role in determining how we perceive and remember our world, and also in decision making (Brosch et al., 2013). Negative emotions interfere with physicians’ clinical decision making (Croskerry & Norman, 2008; Resnick, 2012) and clinical performance (Croskerry, 2014; Schiller et al., 2018). Unprocessed emotions and reactions may also have a negative impact on students’ mental health and on their journey to becoming physicians (Helmich et al., 2014; Monrouxe et al., 2015). Therefore, it is crucial for students to learn how to manage and process negative emotions. The development of abilities to manage emotions can be considered a key factor in the ongoing process of becoming a medical professional (Dornan et al., 2015).

In situations that are emotionally challenging, we sometimes need to regulate our emotions, which involves suppression of feelings as a way of managing them in a certain situation that demands a certain behavior (Doulougeri et al., 2016; Gross, 2002; Lundin et al, 2018; Mann & Cowburn, 2005). The consequences of this strategy in relation to emotionally challenging situations, and on medical students’ learning and how this affect students’ professional identity formation, are relatively unexplored. Considering that junior physicians often feel unprepared for their own negative emotional responses (Lundin et al., 2018), it is important to gain insights into these processes so that students can be given the proper support and be strengthened in their abilities to handle challenging emotions. Considering the result of a recent survey (Sveriges läkarförbund, 2022) showing that approximately one-third of junior physicians in Sweden have serious thoughts regarding leaving their profession, it is of great importance for students, as well as also for society, that we learn more about medical students’ emotional challenges, how they manage them, and in what way faculty can better prepare them for the future.

The aim of the present study was to explore medical students’ experiences related to emotionally challenging situations during work-based learning, their main concerns, the ways in which they resolve them, and what consequences this has in relation to learning and professional identity formation.

### Professionalism and professional identity formation

The theoretical position underpinning this study is the social view of professional identity formation; i.e., the idea that students develop a professional identity through social interactions as they participate in work-based learning (Ahmadian Yazdi et al., 2019; Byszewski et al., 2012; Cruess et al., 2015; Hendelman & Byszewski, 2014; Irby & Hamstra, 2016; Karnieli-Miller et al., 2010; Lave & Wenger, 1991; Scanlon, 2011; Wald, 2015; Wenger, 1998). A part of medical students’ professional identity formation is learning about and developing professionalism. The concepts of professionalism and professional identity formation are elaborated below.

Besides acquiring theoretical and clinical knowledge and skills, students are required to learn about medical professionalism, which is fundamental to clinical practice and an important learning outcome in medical education (Simpson et al., 2002; ABIM, 2003; O'Sullivan et al., 2012; ACGME, 2017). Conceptualizations of professionalism developed over the years and have slightly different foci (Birden et al., 2014; Irby & Hamstra, 2016), where some are primarily concerned with professionalism based on particular virtues and behavior, focusing on certain individual attributes underpinning behaviors and attitudes that are considered to be core traits for the profession. However, they all address aspects of excellence, accountability, altruism, and humanism (ABIM, 2003; Stern, 2005). To achieve a Master of Science degree in medicine, according to the Swedish Higher Education Ordinance (1993:100), students must “demonstrate self-awareness and the capacity for empathy, demonstrate the ability to adopt a holistic view of patients informed by a disciplinary and humanistic approach with special consideration of human rights.” Furthermore, they must “demonstrate the ability to adopt an ethical and professional approach to patients and those close to them, and demonstrate the ability to identify the personal need for further knowledge and undertake ongoing development of their skills.” These requirements are substantially no different from those defined by other medical faculties around the Western world, as for example the UK’s General Medical Council’s “Outcomes for graduates” (General Medical Council, 2018). The concept of professional identity formation is described as a developmental adaptive process in which students internalize certain attributes and roles, resulting in the expression of a physician’s desired behaviors (Cruess et al., 2014). This view highlights the process of becoming a professional, in which students successively grow into the role (Lave & Wenger, 1991; Scanlon, 2011), and the goal is being rather than solely doing. In line with this approach, Cruess et al. (2016) introduced an amended version of Miller’s pyramid, which graphically illustrates this concept. In Miller’s classic article he introduced a four-level pyramid, consisting of knowledge (knows) in the base, followed by competence (knows how), performance (shows how), and finally action (does) at the top of the triangle (Miller, 1990). Cruess et al. added an additional identity—“is”—at the top of the pyramid, illustrating the student’s consistent demonstration of the attitudes, values, and behaviors expected of a physician. At the peak level, students should have incorporated these qualities into their identity (Cruess et al., 2016).

In other words, this journey toward becoming a professional, involving learning about professionalism and developing a professional identity, can be considered as an evolutionary, ongoing, and iterative process (Irby & Hamstra, 2016; Lave & Wenger, 1991; Scanlon, 2011) in which students not only acquire skills and theoretical knowledge, but also absorb approaches related to their future profession. Today we have limited knowledge about how emotionally challenging situations affect medical students’ journey toward becoming physicians, and this study seeks to diminish that knowledge gap.

## Methods

### Methodology

To explore medical students’ experiences in relation to emotional challenges, we chose to use qualitative data: narratives consisting of students’ reflective essays. We aimed to identify students’ main concern in relation to their experiences, as “the main concern highlights the issue or problem that occupies much of the action and attention in the research setting” (Charmaz, 2014). While “grand theories” are linked with all social phenomena and make universal claims assumed to be generalizable to all social groups, cultures, and societies at all times, grounded theory methodology aims to develop “middle-range-theories” (Glaser & Strauss, 1967), which are limited to explaining or understanding a certain social phenomenon in specific social groups or cultures, and thus come closer to actual people’s social actions and meanings in everyday life. To develop a middle-range theory about how medical students experience and manage emotionally challenging situations and how this is related to learning and professional identity formation during their education, a grounded theory methodology was applied. This methodology is a systematic yet flexible method of constructing theory from data, with a focus on social and social psychological processes, such as interpreting and dealing with emotionally challenging situations. In line with constructivist grounded theory, co-construction of data was acknowledged (Charmaz et al., 2018). The research group members consisted of three physicians and medical educators (AL, AS, AW) and three educational researchers (MW, RT, HH). The composition of the interprofessional research team meant increased opportunities for several perspectives to emerge in relation to the data, enabling in-depth analysis and reflexivity.

### Context of the study

The study was conducted in the context of an undergraduate medical curriculum at a Swedish medical university. It is important to note that the data were collected before the outbreak of the COVID-19 pandemic. The Swedish medical curriculum lasts 5.5 years, and there is a clear integration of theoretical knowledge and practical skills. Although basic sciences are the main focus during the first 2 years, students are introduced to the clinical environment as early as their first year, when they spend time at outpatient care clinics. The last 3.5 years of the program consist mainly of clinical courses with workplace learning where students take part in patient care under the supervision of healthcare professionals; in most cases, physicians. These placements rarely last longer than 1–2 weeks at the same ward, and students often meet with several supervisors during a single placement. Professional development is integrated into the formal curriculum, with mandatory components instructing and supporting students to acquire a professional understanding of and approach to their work as a physician, preparing them for their future role. This part of the curriculum includes lectures in ethics, psychology, students’ behavior, leadership, communication skills, and personal development. Students are also assigned to a reflection group consisting of approximately seven students and a group leader, most commonly an experienced physician. The group meets for reflection seminars during the sixth semester. Furthermore, students are assigned a mentor at the beginning of the program: a clinician who they meet once every term for group and individual discussions. Students reflect upon their progress and what they should focus on to continue their professional development. Following the meeting, students document reflections about their professional development in writing.

### Participants and data collection

At the end of the medical program, as part of their final exams, students were required to write a reflective, critical, and analytical essay related to different aspects of their personal development during the program, including their perceived strengths and weaknesses. A considerable amount of time is set aside for the assignment, and students have the opportunity to examine their thoughts thoroughly. As a basis for the essay, students use personal reflections related to their professional development, written iteratively when meeting with their mentor every term. The essay is assessed and scored in terms of reflective abilities, the ability to recognize the nuances and complexity of an event, and the understanding and reflection on one’s own emotions. Students are informed that it is their ability to reflect on their development that is being assessed and scored and not their actual development, per se. For example, if they feel they have not developed at all, they should reflect upon why this is the case, and if the reflection is thorough and multilayered it can theoretically achieve a high score. These reflective essays constituted the data in the study. The first author (AL) sent a letter to all students taking the course over two terms with a request for their participation in the study, including information about its purpose and their rights as participants. Information about the study and a request for participation were made after students had received their results from their final exams, of which the texts analyzed in this study were a part. Informed consent was obtained from all individual participants included in the study. We used students’ explicit answers about their feelings and thoughts about emotionally challenging situations and what they felt they had learned in relation to those situations. It is theoretically an advantage to have the opportunity to explicitly ask students to reflect upon their feelings, as previous research has shown that only 20% of spontaneous narratives have emotional content (Karnieli-Miller et al., 2010). In the part of the essay that is the focus for this study, students were asked to answer and reflect upon the following questions:

During the medical program, you are exposed to situations that can be experienced as emotionally challenging. Has your way of perceiving those situations and your ability to deal with them changed during the time you have attended the medical program, and if so, in what way? Has the program helped you to develop your ability to deal with these situations, and if so, in what way? You are welcome to illustrate your answers with concrete examples.

Out of a population of 282 (158 female, 124 male) students—all of whom participated in the final exams for 1 year, 85 (50 female, 35 male) participated in the current study by giving us their permission to use their reflective essays (Table 1). Students’ choice to take part or not did not affect the passing or failing of exams. Table 1 presents the demographics of the participants. Female students attending the course made up a total of 56%. Approximately the same gender proportion (59%) was seen in the sample. The age range of participants in the study reflected the age range in total. Ethical guidelines concerning research involving humans (Codex, 2019) were followed by all researchers conducting the study. Ethical approval for this study was granted from the Regional Ethical Board (Dnr 2014/1088–31/5).

**Table 1:** Participant demographics

Age	Gender	*N*
23–28	Female	33
	Male	14
29–34	Female	14
	Male	13
35–40	Female	3
	Male	6
41–46	Female	0
	Male	2

### Analysis

In accordance with initial coding as the first analytical coding step within the constructivist grounded theory approach (Charmaz, 2014), all the essays were iteratively read and coded word-by-word and line-by-line by the first author, AL. The initial coding adhered closely to the data, as the author remained open to anything emerging in it. This involved capturing the main meaning of relevant statements or sentences in codes. The codes were derived from the data, not from theory, and the analysis was initially inductive. The aim during this initial step was to focus on actions and processes (Charmaz, 2014; Charmaz et al., 2018). The second author, MW, read about 30% of the 86 documents and coded 10 of them independently in detail to ensure transparency and trustworthiness in the analysis. The constructed codes underwent constant comparative analysis, as the aim was to interpret the data beyond concrete statements (Charmaz, 2014). Some codes were constructed as in vivo codes (Corbin & Strauss, 2008), as students themselves had already theorized certain actions in a commendable way in the texts. As the analysis and the initial codes were constantly compared and grouped, the next step in the analysis was what Charmaz (2014) calls focused coding, in which the most significant and frequent codes from the initial round of coding were compared with each other to guide further analysis and to synthesize large amounts of data into more distilled categories. Interpretations that occurred during the process were written down and saved as memos. Saturation was achieved, since no new concepts were found in the data, when approximately two-thirds had been analyzed (Charmaz, 2014). However, the coding proceeded until all the narratives were analyzed. The categories were then defined and linked to each other by theoretical coding (Thornberg & Charmaz, 2014, 2014), and theory construction was initiated. Analysis was discussed continuously within the research group.

## Findings

Our findings show that students’ main concern related to maintaining an appropriate professional approach in emotionally challenging situations. Here we first describe their main concern, followed by students’ strategies in managing this concern and factors they consider to be facilitators or inhibitors in this process. Thereafter, we describe students’ learning from emotionally challenging situations and present a model illustrating the result; the grounded theory.

### To be professional in emotionally challenging situations

The students’ goals included maintaining empathy, controlling emerging negative feelings, and remaining intellectually focused during the event. In their reported experiences of failed attempts to control their feelings in these situations, resulting in unwanted internal and external emotional expressions, students referred to feelings of shame, decreased communicative ability, and a lack of focus. They described how their aim of achieving a professional approach was motivated not only by the patient’s best interests, but also their own. In the excerpt below, one of the students summarizes his thoughts about what he wants to achieve, describing his aim of being able to manage his feelings in relation to emotionally challenging situations.

The better I get at keeping my desires, feelings, and instincts under control, the better opportunities I will have to develop my empathy and other good attributes which will benefit me and my patients. (student no. 18).

When students were unable to control emerging negative feelings and became overwhelmed by them, they described their lack of control and insufficient suppression of feelings as a negative experience. The following excerpt from another student illustrates this. She describes a situation where she was observing a patient–doctor conversation in which the patient was told that the cancer they had found in her was incurable. The student fought with her emotions, but eventually she started crying.

I remember how I sat on a small chair next to the doctor and the patient lay on the bed roaring and crying. I tried to hold back my tears […] I don’t mean that doctors are forbidden to cry, but I felt I had lost my composure and focus completely. (student no. 14).

While the student actively struggled to suppress overwhelming negative emotions, she did so at the cost of her focus, preventing her from giving the patient in the situation an adequate amount of attention. Thus, when students were unable to manage their emotions, they described difficulties in maintaining their intellectual sharpness and acting in a way that would be beneficial to the patient. Furthermore, students reported that their communication skills suffered when their minds were occupied with the struggle to stay focused in a demanding situation, resulting in a more brusque and imprecise language. This phenomenon could have a negative impact on the outcome of a clinical situation, as communication skills are a fundamental part of a physician’s work and at the heart of professionalism. As feelings overwhelmed students, and they found themselves in situations where they were unable to control their reactions, feelings of shame could arise. One student described an experience where she witnessed the death of a patient and reacted in a way that she felt was inappropriate:

It was an expected death of an 83-year-old lady and yet I became deeply affected. Afterwards I felt ashamed of my selfishness, getting so emotional in front of my supervisor but also in front of peers and the patient’s family. (student no. 73).

The student reacted with shame to what she considered to have been an inappropriate reaction on her part that was not beneficial or supportive for the people around her. The unpleasant feeling of shame made her realize that she was not content with what she considered her inability to manage her emotions in the proper way during the experience. Her reflection of the experience therefore increased her self-awareness.

### Strategies to manage emotions

Our analysis emphasizes that becoming a medical professional involves a struggle related to developing one’s abilities to manage emerging negative feelings in emotionally challenging situations. Managing simply through endurance and failing to acknowledge negative feelings seemed to cause them to linger, and when students perceived that this happened, it often encouraged them to find better strategies to avoid such feelings becoming a burden. In the developmental process of becoming a professional, students had to find their own personal ways of managing emotions adequately. This included actively searching for suitable strategies, depending on situational and personal needs. Students displayed an awareness of the need to solve the obstacles they encountered in this process, and made active and conscious choices regarding suitable managing strategies. The different strategies described by our students were more often used after, rather than during, an emotionally challenging situation.

Experiencing emotionally challenging situations could potentially serve as a catalyst for students to develop management skills that are suitable and beneficial for them. However, some students reported engaging in avoidance. Students adopted several strategies for managing these situations:

#### Reflection with peers, supervisors, and relatives

Students stressed the importance of having the opportunity to reflect together with others after having experienced a situation they perceived as being emotionally challenging. Even if they succeeded in suppressing their emotions during the event, they felt a need to deal with thoughts and feelings afterward. Any lingering questions needed to be answered, if possible. The student quoted in the excerpt below stated that he preferred to reflect with someone he felt safe with or someone who could relate to the situation he had experienced.

In the moment, I experience myself as being very calm and I keep my feelings inside, but I’m actually quite sensitive, so sooner or later I have the need to reflect on what has happened. Usually with a trusted peer. It has also happened that I have talked to supervisors. Preferably it should be someone who is familiar with the situation, a peer or a physician. (student no. 57).

Students also reported reflecting upon troubling situations with relatives and close friends outside the hospital setting, although some of them deliberately spared their families from what they perceived to be the burden of having to listen to their stories, and therefore actively preferred talking to suitable peers and supervisors.

#### Self-reflection

Another way to manage emotionally challenging situations was for students to engage in active internal reflection—reasoning with themselves to find their way, aiming for self-development and a positive outcome of their efforts. One of our students was called out to help in an acute situation after having signed up for a voluntary cardiopulmonary rescue program. The student described a situation where he witnessed the death of a man who had suffered from cardiac arrest in the street. The student experienced a secondary physical reaction afterward, and reasoned with himself about how to relate his experience to expectations from society and the medical profession.

Everything went well; nevertheless, the whole situation felt strange, and I reacted very strongly by shaking and sweating and feeling anxiety a few hours later when I was alone at home. Even if it was a painful experience, I learned a lot from it; taking responsibility in situations can be much more stressful than you expect. […] You have to be prepared to handle situations you can never plan for and be strong when death comes close or into other people’s lives. It’s important that I’m capable of handling my own feelings and thoughts and that I don’t get totally consumed by the profession and what it entails. The reaction I initially defined as being a weakness in the form of a panic attack, in the end proved to be a strength, as I now feel more secure and capable when taking action. (student no. 47).

Our student’s self-reflection helped him to realize that a painful anxiety attack resulting from the acute event was in fact something he believed to be positive, as he had gained experience and self-confidence in knowing he was able to act in an acute situation if necessary.

#### Deliberate exposure

Another active strategy students described was to purposely seek out and expose themselves to potentially emotionally challenging situations. The urge to do so came with the understanding that they would have to be able to manage these situations in the future as professionals, as this is an important and inevitable part of being a physician. Thus, they sought opportunities to gain experience. During these events, they strove to remain active and alert to their own reactions. The following excerpt illustrates a student’s awareness of her own active strategy for exposure.

I still believe that emotionally challenging situations are hard to manage, but today as opposed to a couple of years ago, when I tried to completely block out all feelings, I have learnt that it’s human to react emotionally in such situations and that it might even be considered inhuman to not react at all. I’m going to develop my ability to handle these situations better by exposing myself to them. (student no. 19).

The student negotiates with herself, reflecting on what can be considered an acceptable way of reacting. In addition, she plans for taking responsibility for her continuing development, even if it is likely to be emotionally demanding.

#### Establishing boundaries

Students reported that they made an effort to be mentally present during emotionally challenging situations, to endure even if it was painful for them, and to allow themselves to feel with the patient for the purpose of gaining experience. Students reasoned about the importance of empathy, and on a related note, the importance of setting boundaries. Some of them argued that setting boundaries came instinctively, as an act of self-preservation. Others described it as an active, conscious choice, something that was crucially important to be able to work as a physician. They sought to find a balance between involvement and detachment. As students continued to experience challenging situations, they seemed to develop ways of finding this balance and abilities that helped them better endure emerging emotions. In the excerpt below, one of the students reflects on how she has become more accustomed to managing her feelings; even though she still experiences negative reactions internally, she now feels that she is better equipped to handle them.

One goal is to retain my empathy and to cut off too many personal feelings at the same time if needed. I think I have gradually hardened during my education, but I can still experience a certain amount of anxiety and a wish to escape when confronted with an emotionally challenging situation—for example, when meeting a deeply depressed patient. However, now I succeed in remaining strong and confident in most situations. (student no. 78).

The student shows awareness that she still needs to develop toward a higher state of professionalism, noting that she is now able to handle “most” situations with confidence, with the inferred meaning that this does not yet apply to all situations.

#### Avoidance

Some of the students reported how they use avoidance as a strategy to manage emotionally challenging situations, although this might entail missing out on valuable learning opportunities. In the quote below, a male student describes his way of avoiding encounters with what he found to be difficult patients. He talks about how he hopes to address the issue in the future, but at present does not have appropriate or effective tools to solve the problem.

Situations I have perceived as very difficult, include meeting unstable patients and those with psychiatric conditions. I think it’s hard to manage situations with people who are unpredictable or aggressive and it has led me to try to avoid such situations quite often, even if I should practice them. It might be something to do with me being a quiet sort of person resulting in a lack of authority that certain situations demand. This is something I know I will have to fight against in order to provide good and safe medical care. (student no. 28).

The student thus postponed managing certain situations, missing out on important experiences and risking the inhibition of his professional identity formation.

### Factors that facilitate or inhibit

According to the students’ reports, some factors were particularly influential for developing their abilities to manage emotionally challenging situations, and students described facilitating as well as inhibiting factors that affected the process. They emphasized the importance of debriefing close to the event and in an environment where they felt safe, preferably with trusted peers, and supervisors they believed to be good, attentive role models. These kinds of ideal circumstances were believed to facilitate management.

The absence of debriefing and perceived negative role models were described as an inhibiting factor for managing emotional experiences. In the following excerpt, a student describes his feelings after having experienced an acute event with a fatal outcome for the patient, and the lingering negative effect this experience had on him.

It was a situation where I as a medical student went home after a tragic cardiac arrest in a young, previously healthy patient where I believed there had been mistakes in the treatment of the patient. Maybe it was adequately performed, but when there is no room for discussion and no possibilities to ask questions, it creates guilt, lack of trust, and unpleasantness. (student no. 32).

This student experiences how a lack of adequate debriefing and supervision may complicate students’ development and negatively affect their self-efficacy. The failure of supervisors to legitimize students’ feelings as appropriate in relation to an emotionally challenging event seemed to inhibit students’ ability to manage. One student reported how he perceived a situation where emotionally difficult circumstances were not acknowledged as such by supervisors, and the lack of support left him with an unpleasant feeling:

During anatomy classes, the teachers were very aware that the situation had the potential to be emotionally difficult, and proceeded cautiously. They started by showing just an arm, and then carefully unveiled the bodies, one part at a time, which you then worked with. This made it easier to handle. A few terms later, we were placed in the autopsy department, and immediately it was tougher. Here, it wasn’t just people who had died of old age or who had chosen to donate their bodies; now there were ordinary people who had died just a few days or hours ago and now lay there cut open on the tables. […] I think that teachers’ understanding had decreased. At this point we were just expected to be able to manage this. I don’t think they understood the difference I experienced. The latest example was recently during the course in forensics. The understanding that you might feel discomfort was then almost zero […]. So concerning this, I think medical school was bad at preparing or training us for emotionally challenging situations. (student no. 31).

Examples were also given where role models had a direct and negative impact on how students managed an emotionally challenging situation. The following excerpt highlights the negative effect role models can have, whereby the student incorporated the negative values presented to her, which is arguably counterproductive to a positive professional identity development.

Eventually, as I have heard more and more conversations in countless coffee rooms about difficult patients and patient categories, and after having a supervisor turn to me for the tenth time saying “Don’t you think he just wanted a medical certificate?”, it has become hard to defend yourself against the cynical attitude expressed by many health care professionals. And all of a sudden you discover that you yourself are thinking that here comes a patient belonging to the category “difficult patients,” and you start wondering how and when you started thinking like that. (student no. 11).

Furthermore, being a member of the team and having the opportunity to contribute during a challenging situation appeared to make the experience easier for students to manage. One student described a situation with a fatal outcome. In spite of the negative outcome for the patient, his perception of belonging resulted in a positive experience in contrast to his earlier negative experiences in similar situations.

The bleeding was so extensive that it couldn’t be stopped and after a while we had to stop. Afterwards I was totally empty, nothing strange about that, but as I had been active and felt that I had actually contributed in the situation, it had a purpose, or rather I felt a sense of belonging that produced totally different feelings. I chose not to go straight back but participated in the anesthesiologists’ debriefing, where I got positive feedback for being able to work in the stressful situation. Afterwards I took a break to unwind before I went back to my placement. (student no. 24).

Considering the fact that students’ placements at outpatient clinics were short, often not exceeding 1 week, students’ opportunity to become a member of the team decreased, and students reported that “being new at work” took continuous effort. This seemed to be an inhibiting factor in relation to having good opportunities to manage emerging negative feelings arising from unpleasant experiences, as illustrated by the following quote:

As a student, I’ve felt compelled to be accepted by a person or a group before I dared to take part in the collaboration. […] It has been a huge strain on me, wanting to be accepted in a group and constantly having to change context, placement, study groups, etc. back and forth […] I’ve been so tired and socially exhausted that I haven’t had the energy to take care of my relationships outside the school or hospital. As peers we haven’t had the energy to support each other much either in this matter, as we have all been equally tired because of all the different social contexts. (student no. 34).

### Learning from emotionally challenging situations

Students’ experiences of emotionally challenging situations, alongside their aims to achieve and maintain a professional approach related to these experiences, resulted in an internal struggle that created a need to adequately manage their feelings, both during and after the experience. During this process, students gained insights about different aspects of themselves. They became more aware of their own reactions, which had the potential to lead to self-knowledge about their shortcomings, resources, and personal needs. Thus, there seemed to be valuable learning opportunities related to exposure to emotionally challenging situations.

#### Awareness of personal reactions

Students’ awareness of their own personal reactions is an important factor, providing the opportunity to take better precautions when facing similar challenges in the future. For example, students described being aware of how certain patient-related situations provoked a physical reaction. Others reported an awareness of the risk of identifying with patients and how this could cause a strong psychological reaction. One student described how she had not been able to stay in a particular situation because of her strong emotional reaction due to identifying with patients.

The toughest situation I’ve come across was during my placement at the neonatal ward when I was pregnant myself. It became too difficult to see sick or premature children, born earlier or in the same week as my unborn child. There I lost my professional approach and I related to my own child. It ended with me walking away from the situation so as not to cry in front of the children or their relatives. I think I cried for a whole day. But then, after that incident, things have gone better. It was truly an awakening, [to] the fact that you can’t relate everything to yourself. It gets too hard. (student no. 75).

The student described the experience as an awakening, as she realized why she had been taking things so personally, and that she needed to develop a strategy to protect herself.

#### Awareness of personal needs

It is important for students’ futures as professionals to be aware of what kind of support is necessary for them, as they will face emotional challenges during their careers that may have a negative effect on their well-being if not taken care of properly. Students emphasized the need for debriefing, and also what happens if debriefing opportunities are absent.

If I get the opportunity to discuss with colleagues and go through the event, the situation doesn’t need to affect me negatively. On the other hand, if I try to put a lid on it and keep my feelings aside and just move on, it creates big problems for me. The event feels unfinished and worries me for a long time. (student no. 8).

This student reported experiencing lingering negative thoughts when he did not give himself the opportunity to reflect upon an emotionally challenging event.

#### Awareness of need for improvement

Students described awareness of areas where there was a need for personal improvement. One of the students discussed what she considered to be a shortcoming in her personality that hindered her from being fully professional in relation to patients.

This desire to be liked also applies to my meetings with patients. Nothing could be as satisfying as a content patient who feels that he or she has been listened to, who has got what she or he wanted. Now, I have to find a good balance as I start working. Standing up for myself when it comes to my medical knowledge that I should have by now. (student no. 34).

Stress due to time pressures was a significant challenge in relation to maintaining a professional approach, as students found it challenging to focus on displaying empathy toward patients under these circumstances. This phenomenon is illustrated by the following excerpt, in which the student contends with the fact that lack of time could jeopardize his empathetic approach, with an awareness of the importance of finding a solution to this dilemma. Students found it dissatisfying when they were not able to focus on being empathetic.

When I’m stressed, I notice that I don’t have the energy to be empathetic, because then time is the first thing on my agenda. I’ve thought a lot about how I need to work on my stress—to allow myself to be behind in the schedule but have satisfied patients, and above all to be satisfied with my work myself, rather than stressing and providing insufficient patient contact. (student no. 25).

Being able to focus on several things at once is an important skill when meeting with patients, otherwise the physician is at risk of missing out on crucial information. Focusing solely on finding the correct diagnosis, for example, risked occupying students’ attention at the cost of being empathetic. This contributed to making it harder to actually solve the problem at hand, something one of the students highlighted in the following description of a situation where he was fully focused on solving what he thought was the problem, and not listening to the patient.

During my clinical placement I met a young female patient who had been on sick leave for over a year because of burnout, and now she had come in because of pain in the wrist. I had recently attended a seminar about mental disorders manifesting as pain. I asked her several questions about her mental health problems and wasn’t at all responsive to her requests not to address this during the consultation. It ended with her getting angry with me and I lost my composure and therefore my focus. It wasn’t a good meeting. If I had illustrated a higher level of empathy, I would have realized this wasn’t the moment to deal with her psychosocial disorders. Most likely, my empathetic skills were displaced because of my simplistic aim to diagnose. The empathetic skill is for me an effort that competes with other effort and it’s not always empathy that comes out victorious. (student no. 18).

The student’s non-responsive attitude made it difficult to reach and help the patient adequately, and as a result the situation got out of hand. He realized he had not acted professionally as he reflected on the situation afterward.

#### Awareness of the risk of detachment

Students paid attention to the risk of developing a detached attitude toward patients, which was considered undesirable, and they expressed concerns about developing hardness as a result of their experiences. They hoped that awareness and their attention of this phenomenon would help them to preserve their empathetic skills. One of the students discussed the risk of “burning out” his empathetic capacity.

During my education I have met with and witnessed many human fates, which have helped me gain experience about how life can be. At the same time, I can see the risk of reducing my empathetic ability. (student no. 20).

Another student described her awareness of the difference between empathy and sympathy, something she had developed over time, and how this had been helpful to her. Furthermore, she reported on how she had realized that letting things get to her emotionally was not sustainable in the long run.

I have to a larger extent started to separate empathy from sympathy. I think it’s a great number of meetings with patients in challenging situations during my education and by work outside of education that have contributed to this. Maybe it’s impossible to feel a cutting pain right down to the bones every time something sad happens, but I feel that I still have empathy for human beings preserved to the same extent. To be able to distinguish between empathy and sympathy might be the strategy that’s needed in order for a doctor not to go under. And I think it can be a part of development towards a professional approach. (student no. 14).

### A grounded theory of medical students’ professional identity formation through emotionally challenging situations

Medical students experience emotionally challenging situations during clinical practice. Strong emotions that emerge in relation to these events must be addressed, both during the event as well as afterward. Our results show that students often forced themselves to suppress strong negative feelings during such situations, in an attempt to maintain a professional approach toward patients and medical staff in every situation, no matter what. This has been described as producing an internal struggle, a struggle that could take up a great amount of students’ strength and focus as they tried to control their reactions. When students failed to suppress their feelings, allowing them to take over, they perceived their own behavior as unprofessional and not beneficial either for the patient or for themselves. Feelings of shame could be the result. Lingering negative feelings were common; students felt a need to deal with these feelings and actively sought ways of doing so. In this way, emotionally challenging situations may be viewed as a valuable opportunity, a catalyst in students’ development and professional identity formation. As they searched for ways to resolve their emotional dilemmas, students encountered both facilitating and inhibiting factors. Opportunities for internal and external reflection were considered necessary. Furthermore, good role models were perceived as important. When students found themselves in a meaningful position and felt like part of a team, their experiences were more likely to be described in a positive way even if the outcome for the patient had been fatal. This insight illustrates the importance of participation.

**Fig. 1:**
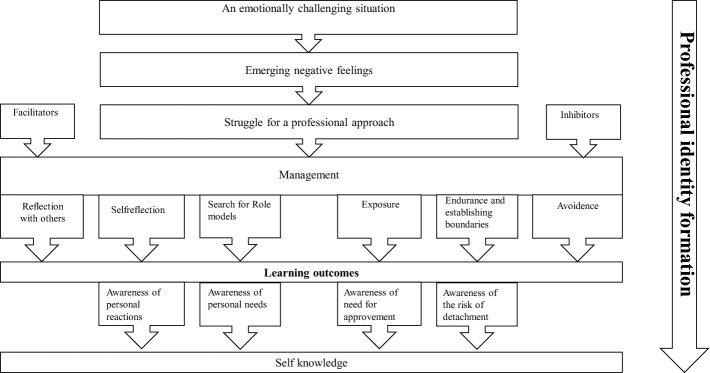
A grounded theory of medical students’ professional identity formation through emotionally challenging situations

The study shows that emotional challenges could serve as important learning experiences. As students iteratively experienced and became aware of their reactions and behaviors, they seemed to become better able to manage their emerging negative feelings in similar situations with decreasing levels of effort. Learning related to emotionally challenging situations seemed to be closely linked to professional identity formation. The iterative process of experiencing difficult events, combined with the drive to achieve a professional approach, resulted in an increasingly improved ability to manage negative feelings. Students’ learning were related to self-awareness and resulting self-knowledge. This developmental process is an ongoing iterative process and an important part of professional identity formation (Fig. 1). Thus, emotionally challenging situations can play a crucial role as a catalyst in students’ development in becoming professionals.

## Discussion

Our findings show that emotionally challenging situations during medical students’ work-based learning can cause an internal struggle as students, regardless of the emotional magnitude an event evokes, strive to maintain a professional approach in every situation that arises. Students’ goal can be summarized as the capability to focus on what is in the best interests of the patient, with the goal to be able to act professionally in every situation, “by doing the right thing at the right time, and by doing it in the right way and for the right reason” (Iserson and Egan, 2019). When the students in our study perceived that they failed in their attempts to be professional in an emotionally challenging situation, and they were unable to meet their own and others’ expectations, they reported feelings of shame and inadequacy. In their effort to resolve the issue of emerging conflicting feelings, students used several strategies for managing these. They engaged in self-reflection internally and with peers and supervisors. Self-reflection is known to be at the heart of students’ professional identity formation (Wald, 2015) and generally important for well-being (Harrington & Loffredo, 2010; Niemi, 1997; Ouliaris, 2019; Selwyn & Grant, 2019). Students reflected on the importance of finding the balance between distance and closeness in relation to patients, and establishing boundaries so that unpleasant feelings would not get to them. Some students deliberately exposed themselves to emotionally challenging situations, whereas others reported on how they engaged in a possibly more undesirable avoidance behavior, missing out on experience and possibly delaying their professional development. A study by Schiller et al. (2018) concluded that certain strategies of managing emotional challenges, such as distancing, avoidance, and keeping their feelings to themselves, increased during medical students’ clinical years, and these strategies had a negative impact on academic performance. Furthermore, feelings left unattended can eventually cause students to continue to distance themselves, with the risk of detachment or cynicism (Helmich et al., 2014). In our study, we found that suppression of emotions could cause them to linger. Thus, students had to deal with their emotions and make sense of them to feel better. In line with this, earlier research has shown that emotions evoked from situations generating moral distress can continue for many months after an event (Monrouxe et al., 2015).

On their journey to becoming professionals, students have to gain experience and find their own path in terms of how to manage emotional challenges. Our study indicates that through this process, students gained learning insights about themselves, such as an awareness about their reactions in different situations and their personal needs to endure, their need for improvement, and an awareness of the risk for detachment. Thus, emotionally challenging situations have the potential to enhance students’ self-awareness, acknowledged to be of importance in relation to medical students’ development (Dobie, 2007; Novack et al, 1999) and subsequently self-knowledge. Furthermore, the results suggest that parallel to their increasing self-knowledge, students took leaps in their development toward a professional identity. This is illustrated by the fact that, when later challenged in similar situations, the students seemed to be better prepared and found it easier to maintain a professional approach in relation to the emotionally challenging situation. Students’ self-awareness of their feelings and reactions related to emotionally challenging situations is likely to have a positive impact on the future transition to working as physicians.

Thus, supporting students’ professional identity formation goes beyond explicitly teaching professionalism (Cruess et al., 2019), and our findings that emotional encounters influence professional identity formation are supported by a study by Wald et al. (2019). If students are given proper guidance and support, as we saw in our study, supported by earlier studies (Sarraf-Yazdi et al, 2021), there could be gains to be made related to their professional identity formation, with the goal of obtaining an education that meets the expectations of teaching students not only to be skilled professionals, but also how to endure difficult situations, develop and preserve humanistic qualities, and develop their resilience, as well as the process of adapting well when facing adverse events and stress and the ability to bounce back (Epstein & Krasner, 2013; McCann et al., 2013; Wald, 2015).

The recent COVID-19 pandemic, with the tremendous pressure on healthcare professionals, underpinned the emotional dimensions of the profession and the obvious importance of physicians’ psychological robustness and resilience. This highlights the absolute need for medical students to be prepared for emotionally challenging situations when they take on their future role as physicians.

Our conclusion is that emotionally challenging situations should not be avoided, but instead serve as valuable and irreplaceable learning opportunities, and students should be encouraged to face them. Nevertheless, students need proper support when facing these situations. Our results show that negative role models and a non-supportive clinical environment during work-based learning may contribute to students managing their negative feelings in an inadequate manner, leading to maladaptive responses and negative outcomes. This highlights the importance of giving students and supervisors opportunities to interact over time, building trusting relationships that are fundamental for learning from a sociocultural perspective (Hägg-Martinell et al., 2017). The possibilities of establishing more profound relations with patients as well as supervisors made students feel better prepared regarding facing ethical dilemmas, and an increased professional development was reported (Bates et al., 2013; Brown et al., 2022; Cruess et al., 2019).

Our findings corroborate earlier research suggesting that students can be better prepared for the emotional aspects of clinical practice and be offered better suited and more relevant support (Bore et al., 2016; Steinauer et al., 2019; Wald, 2015; Weurlander et al., 2018, 2019). Apart from establishing professional identity formation as an educational objective and promoting faculty development to ensure understanding of the concept, students themselves need to be engaged in their own development, and supervisors could facilitate by assisting them in charting their progress. In conclusion, our findings suggest that emotionally challenging situations play a crucial role in medical students’ professional identity formation, underpinning the importance to seize valuable and unique opportunities for students to learn in relation to these situations. Highlighting the role of emotional challenges in medical education is an important contribution, as it may enhance insights and open up possibilities to support students in their process of achieving a positive professional identity formation.

## Limitations

Self-reflection has become an established part in health profession education (Mann et al., 2009) and is integrated in the curriculum at Swedish medical universities. Even though medical students are trained in self-reflection, a risk of re-authoring their experiences is a possible consequence of the lapse of time (Carver & Connor-Smith, 2010).

As our data were part of students’ final exams, one could argue that this might have an impact on students’ descriptions of their experiences and development. However, students were informed and asked about participation after they had received the results from their final exams. They were also clearly informed before the exams, orally and in writing, about how their essays were to be assessed. To pass, the students must be able to reflect, analytically and critically, on their experiences. The examiners did not assess students’ professional development per se, but the capacity to reflect. Thus, students reporting on poor development would pass the exam if they presented a thorough and critical analysis on why this was the case. This approach may have facilitated students’ honest stories. Nevertheless, there might be a risk of students’ trying to adapt to what they thought was expected of them. From our point of view, it seemed likely that students did not avoid describing and reflecting upon personal failures and problems in their reflective essays.

As our data were composed of written narratives, a limitation of the study was not being able to ask follow-up questions to enable a deeper understanding of students’ experiences. However, we found a majority of the essays to be nuanced, with students taking multiple aspects into consideration when reflecting.

As the study was performed at a single university, transferability of our findings is dependent on similarities with other contexts.

### Implications for medical education

Emotionally challenging situations, if acknowledged by faculty and supervisors, can be a valuable and irreplaceable learning source for medical students, as such experiences seem to push students in the direction of professional identity formation. The iterative process is crucial to professionalization, allowing students to gain from experience and achieve successively increased ability to think and behave in the best interest of the patient. Faculty need to be aware of students’ experiences so that opportunities for learning in relation to emotionally challenging situations can be improved. To be able to talk to supervisors and peers close to an emotionally challenging event is crucial, but lack of time in clinical practice is often an issue. Interactive reflective writing is known to play an important role in enhancing students’ emerging professional identity formation (Wald et al., 2019). To continuously support and encourage students’ reflective writing throughout medical education, emphasizing the importance of it in relation to self-awareness and personal and professional development, must be considered important. If students realize the value of active reflection, there is a good chance they will continue doing it and make it a habit as they face the realities of being junior doctors and become resilient professionals aware of their need for continuous development. Having access to facilities where students can meet with peers and discuss and reflect upon their experiences in an unconstrained manner is valuable. Longer placements in the same clinical setting also appear to be important, enhancing students’ feelings of being a member of the team, contextualizing their experiences, and enabling questions to be answered by experienced supervisors. Furthermore, faculty and supervisors should be aware of the important learning opportunity represented by these emotionally challenging situations, and how they can contribute to students’ development during their professionalization journey. Students must learn how to handle feelings related to emotionally challenging situations to become physicians, as this is crucial for maintaining a sustainable career. The importance of emotions in medical education deserves more attention and recognition.
